# The Design and Implementation of a Dynamic Measurement System for a Large Gear Rotation Angle Based on an Extended Visual Field

**DOI:** 10.3390/s25123576

**Published:** 2025-06-06

**Authors:** Po Du, Zhenyun Duan, Jing Zhang, Wenhui Zhao, Engang Lai, Guozhen Jiang

**Affiliations:** 1 School of Mechanical Engineering, Shenyang University of Technology, Shenyang 110870, China; dupo@sut.edu.cn (P.D.); zhangjing_8906@sut.edu.cn (J.Z.); zhaowenhui@sut.edu.cn (W.Z.); 2 Engineering Training Centre, Shenyang University of Technology, Shenyang 110870, China; lai@sut.edu.cn (E.L.); jiangguozhen@sut.edu.cn (G.J.); 3 School of Mechanical Engineering, Liaoning Mechanical & Electrical College of Technology, Dandong 118009, China

**Keywords:** binocular vision, rotation angle measurement, calibration, coordinate transformation, measurement accuracy

## Abstract

High-precision measurement of large gear rotation angles is a critical technology in gear meshing-based measurement systems. To address the challenge of high-precision rotation angle measurement for large gear, this paper proposes a binocular vision method. The methodology consists of the following steps: First, sub-pixel edges of calibration circles on a 2D dot-matrix calibration board are extracted using edge detection algorithms to obtain pixel coordinates of the circle centers. Second, a high-precision calibration of the measurement reference plate is achieved through a 2D four-parameter coordinate transformation algorithm. Third, binocular cameras capture images of the measurement reference plates attached to large gear before and after rotation. Coordinates of the camera’s field-of-view center in the measurement reference plate coordinate system are calculated via image processing and rotation angle algorithms, thereby determining the rotation angle of the large gear. Finally, a binocular vision rotation angle measurement system was developed, and experiments were conducted on a 600 mm-diameter gear to validate the feasibility of the proposed method. The results demonstrate a measurement accuracy of 7 arcseconds (7”) and a repeatability precision of 3 arcseconds (3”) within the 0–30° rotation range, indicating high accuracy and stability. The proposed method and system effectively meet the requirements for high-precision rotation angle measurement of large gear.

## 1. Introduction

In the field of precision metrology, angle measurement technology plays a pivotal role, particularly in industrial applications, such as aerospace equipment manufacturing, precision machining, and high-accuracy quality inspection, where the precise measurement of component rotation parameters holds significant value [1,2]. Modern industry imposes two core requirements on this technology: first, the need to achieve micrometer-level measurement accuracy and, second, the demand for intelligent automated measurement [3]. Depending on the measurement principles, current angle measurement technologies can be primarily categorized into four types: contact-based measurement relying on mechanical transmission principles [4,5,6], electromagnetic induction-based measurement [7,8,9,10], optical interference-based measurement [11,12,13], and machine vision-based measurement utilizing digital image processing [14,15,16,17,18]. Among these, the first two contact-based methods are susceptible to environmental interference, such as temperature, humidity, and vibration. Although optical measurement can achieve sub-micrometer accuracy, its measurement field of view is limited, and the optical system setup is complex. In recent years, thanks to advancements in computational processing power and the widespread adoption of high-resolution industrial cameras, non-contact measurement techniques based on machine vision have demonstrated significant advantages [19,20]. These methods combine high measurement accuracy, excellent stability, and strong dynamic measurement capabilities [21,22].

For machine vision-based measurement of component rotation angles, in [23], the authors introduced a monocular vision-based pose measurement method using cooperative targets, where a circular planar target and feature point extraction algorithm were designed, combined with RANSAC and topological fitting optimization, to achieve pitch and roll angle measurements. In [24], the authors presented a non-contact angle measurement approach based on rotating spot images and machine vision, utilizing elliptical trajectory fitting for angle detection. In [25], the authors proposed a high-precision monocular vision-based rotation angle measurement method that employs coordinate rotation equations to determine the rotation angle of calibration plates. In [26], the authors adopted a non-contact measurement method based on machine vision technology to detect winding tilt angles, conducting a comparative study using an improved interval rotation projection method, a quadratic iterative least squares method, and the Hough transform detection approach. In [27], the authors proposed a machine vision-based method for measuring and controlling brush angles, obtaining data on formed angle values and springback angle values using computer vision and image processing algorithms. In [28], the authors designed a dedicated marker and a novel measurement algorithm based on this marker in a monocular vision measurement system. By identifying auxiliary reference lines that can reflect azimuth angle information in real time, they derived a pitch angle calculation formula through mapping relationships. In [29], the authors developed a machine vision system for real-time measurement of fiber angles on the surface of radially braided preforms, enabling precise angular measurement for preforms with varying diameters. The proposed image analysis algorithm achieves automated angle measurement through edge detection and autocorrelation techniques.

Neuromorphic vision sensors offer a novel approach for dynamic measurement. In [30], the authors utilized retinal neural spike data to quantitatively assess decoded visual stimuli through a neural network decoder, establishing a comprehensive decoding evaluation framework incorporating six image quality assessment metrics. This study successfully achieved the decoding of dynamic visual scenes from retinal spike signals. In [31], the authors propose a line-based pose tracking method for uncooperative spacecraft utilizing a stereo event camera. Additionally, they constructed a stereo event-based uncooperative spacecraft motion dataset encompassing both simulated and real events. These studies have pointed out directions for future research. However, considering the reliability requirements of measurement systems in industrial settings, this study adopts an industrial camera solution with an extended field of vision.

The current monocular vision-based angle measurement methods are significantly limited in high-precision inspection of large-scale components, particularly in the high-accuracy measurement of large gear tooth profiles. First, there is the issue of geometric errors caused by large-field-of-view distortion. Monocular cameras exhibit significant radial and tangential distortion in wide fields of view, causing edge feature point coordinates to deviate from their true positions [32]. This is especially pronounced when using wide-angle lenses, where distortion at the image edges can be 3 to 5 times greater than in the central region, leading to nonlinear accumulation of angle calculation errors. Second, edge resolution degradation and feature extraction failure become problematic. As the size of the measured object increases, the physical dimension represented by each pixel also grows significantly [33]. For example, when measuring a 2 m-diameter gear, a monocular system covering the full field of view may experience edge resolution dropping to just one-tenth of that in the central region, resulting in sub-pixel blurring of critical tooth profile features. Optical measurement faces several limitations in large-scale component angle measurement. Line-of-sight occlusion is a major challenge, particularly in complex structures where self-occlusion occurs in angular regions, such as internal right angles. This often necessitates the use of multiple sensors or repeated repositioning, significantly increasing system complexity [34]. For large parts, dimensional effects amplify error propagation, typically requiring supplementary laser tracker measurements for global datum control to ensure accuracy [35,36]. Additionally, surface characteristics affect measurement results. When surface roughness (Ra) is below 0.2 μm, high reflectivity can introduce specular reflection noise. While anti-glare sprays are commonly used in engineering applications to mitigate this, they typically introduce an additional 15–20 μm of measurement error.

In the field of gear measurement, gears with a module ≥10 mm or a pitch circle diameter >500 mm are typically defined as large gears. The in-machine measurement accuracy of large gear tooth profiles based on the meshing method is primarily determined by gear rotation angle errors and meshing line errors [37]. Tooth profile measurement requires a high-precision angular reference. The tooth profile error specified in gear accuracy standards is calculated as a linear value along the meshing line direction. As the diameter of the measured gear increases, the linear error also increases accordingly, leading to a decline in angular measurement accuracy, which is highly unfavorable for tooth profile measurement of large-diameter gears [38,39].

To address the aforementioned issues, this study proposes an extended field-of-view measurement method based on binocular collaborative vision. This method innovatively employs a high-precision measurement reference plate as a unified spatial reference for the binocular vision system, aligning the fields of view of both cameras to the coordinate system of the measurement reference plate. In the specific implementation, the binocular camera system is first rigorously calibrated to precisely calculate the physical coordinates of the two camera centers within the measurement reference plate’s coordinate system. The binocular system synchronously captures image data of the measurement reference plate, and image processing algorithms are used to extract the coordinates of the two cameras’ field-of-view centers on the reference plate. The spatial positional relationship between these two points is then fitted. When the measured large gear rotates, the connecting line between the two camera centers rotates accordingly. By tracking the angular changes of this connecting line in real time, the rotation angle of the large gear can be accurately calculated. Leveraging the spatial complementarity of the dual cameras, the measurement range is effectively extended through field-of-view superposition, enabling high-precision measurement of large gear rotation angles.

## 2. Basic Theory

**Figure 1:**
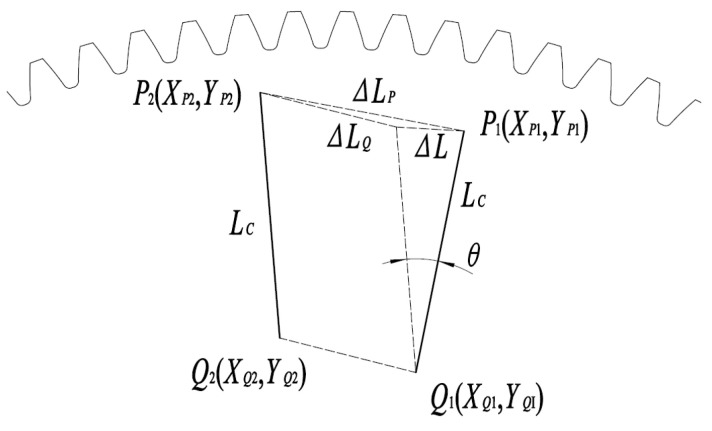
Schematic of rotation angle measurement.

In the gear coordinate system (i.e., the measurement datum plate coordinate system $O_{4}(X^{\left(4\right)},Y^{\left(4\right)})$), the pre-rotation field-of-view centers of the binocular camera are $P_{1}(X_{P1},Y_{P1})$, $Q_{1}(X_{Q1},Y_{Q1})$, while the post-rotation field-of-view centers become $P_{2}(X_{P2},Y_{P2})$, $Q_{1}(X_{Q2},Y_{Q2})$. See Figure 1.

The displacement $∆L_{P}$ and rotation angle $\delta_{P}$ between the first and second output images from Camera 1 are given by the following:

(1) $$∆L_{P}=\sqrt{{{(X}_{P2}-X_{P1})}^{2}+{{(Y}_{P2}-Y_{P1})}^{2}}$$

(2) $$\delta_{P}=arctan\frac{Y_{P2}-Y_{P1}}{X_{P2}-X_{P1}}$$

The displacement $∆L_{Q}$ and rotation angle $\delta_{Q}$ between the first and second output images from Camera 2 are given by the following:

(3) $$∆L_{Q}=\sqrt{{{(X}_{Q2}-X_{Q1})}^{2}+{{(Y}_{Q2}-Y_{Q1})}^{2}}$$

(4) $$\delta_{Q}=arctan\frac{Y_{Q2}-Y_{Q1}}{X_{Q2}-X_{Q1}}$$

The chord length ∆L can be calculated using the Law of Cosines as follows:

(5) $$∆L={∆L_{Q}}^{2}+{∆L_{P}}^{2}-2∆L_{Q}∆L_{P}cos(\delta_{Q}-\delta_{P})$$

Since the baseline distance $L_{C}$ of the stereo camera has been calibrated, the rotation angle θ can be calculated as follows:

(6) $$\theta =2arcsin\frac{∆L}{2L_{C}}$$

To improve the measurement accuracy of rotation angles, the length of line *PQ* must not be excessively short. When measuring the rotation angle of line *PQ* using a vision system, the camera’s field-of-view must encompass not only the two endpoints of *PQ* but also the entire trajectory from its initial to final rotational positions. Employing such a large field-of-view compromises the measurement precision of a single camera. Consequently, this paper proposes a binocular camera system to achieve high-precision rotation angle measurements for large gear.

## 3. System Solution Design

### 3.1. General System Design

**Figure 2:**
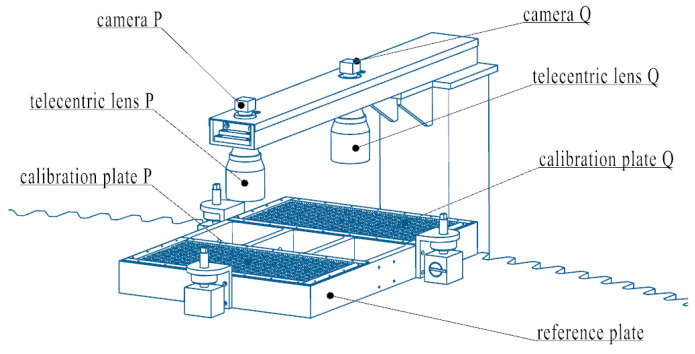
Rotating angle measurement system for large gear.

The binocular vision rotation angle measurement system primarily consists of CMOS industrial cameras, dual telecentric lenses, a measurement reference plate, and two 2D dot array calibration plates, as illustrated in Figure 2. The measurement reference plate is fixed on the large gear and rotates with it. Using the 2D dot array calibration plate as a feature marker, the binocular cameras continuously capture images of the measurement reference plate on the rotating large gear. The rotation angle value is obtained by calculating the coordinates of the binocular cameras’ field-of-view center points within the measurement reference plate coordinate system.

### 3.2. Measurement Reference Plate Design

**Figure 3:**
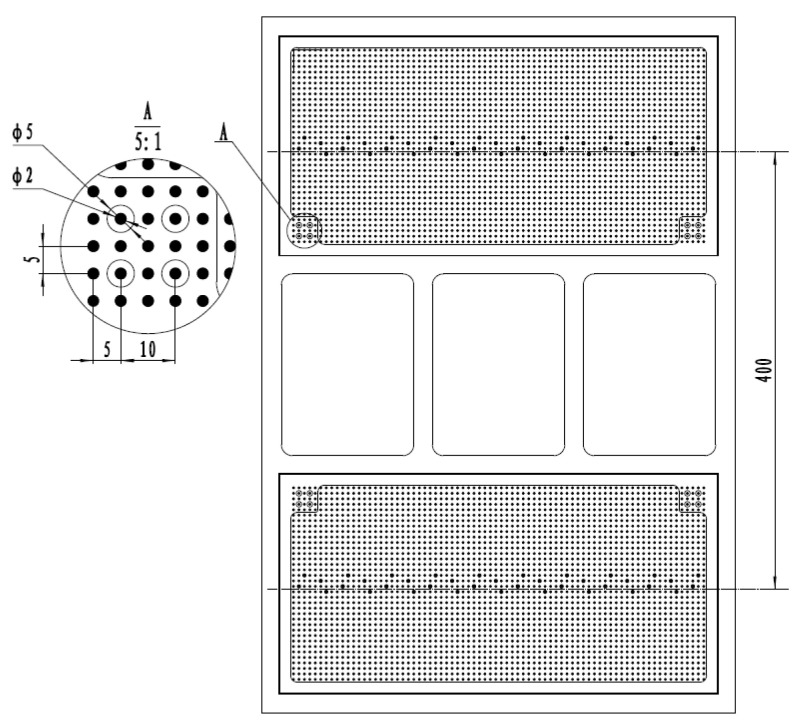
Design of reference plate.

The measurement reference plate serves as the mounting base for two parallel 2D dot array calibration targets. Each target comprises a 36 × 76 orthogonal grid of calibration circles with 2 mm diameter and 5 mm pitch. The centers of the two calibration plates are separated by 400 mm on the reference plate. Sixteen high-precision alignment holes are distributed across the plate’s structural framework to establish coordinate transformations between the calibration plates and the reference plate. These holes provide metrological traceability by bridging the coordinate systems of individual calibration plates with the unified reference frame, as illustrated in Figure 3.

### 3.3. Measurement Reference Plate Calibration Method

#### 3.3.1. Coordinate System Establishment

**Figure 4:**
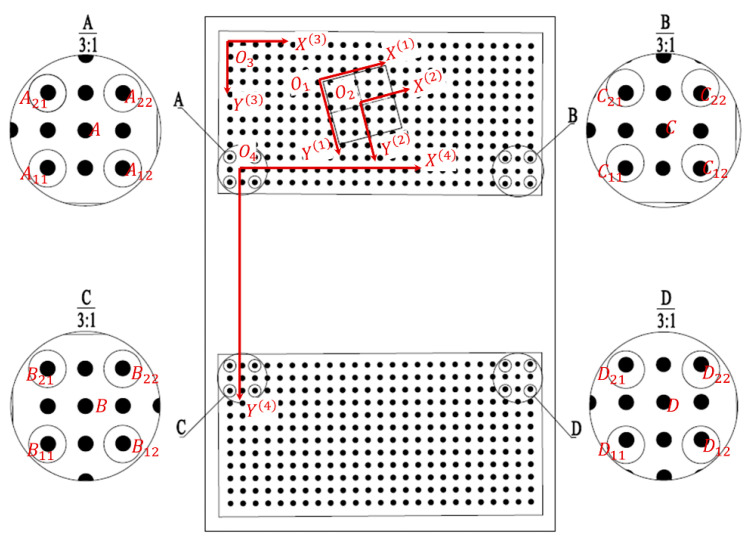
The relationship between coordinate systems.

In the rotation angle measurement system, multi-level coordinate system conversion demonstrates clear engineering necessity. From the perspective of rotation angle measurement principles, the measurement essentially requires establishing precise correspondence between image pixel space and real-world coordinate systems, a process that inherently involves coordinate conversion. While traditional monocular vision solutions typically adopt a three-level conversion architecture, our binocular measurement system faces special engineering constraints: large-sized 2D dot array calibration boards present practical challenges, including difficult-to-guarantee machining accuracy, significantly increased accumulated errors, and prohibitively high manufacturing costs. To address these issues, this study innovatively employs a dual 2D dot array calibration board splicing scheme, consequently introducing an additional fourth-level coordinate conversion step. This involves conversions among four distinct coordinate systems: image pixel coordinate system $O_{1}(X^{\left(1\right)},Y^{\left(1\right)})$, image physical coordinate system $O_{2}(X^{\left(2\right)},Y^{\left(2\right)})$, 2D dot array calibration board coordinate system $O_{3}(X^{\left(3\right)},Y^{\left(3\right)})$, and measurement reference board coordinate system $O_{4}(X^{\left(4\right)},Y^{\left(4\right)})$. The relationships between these four hierarchical coordinate systems are illustrated in Figure 4. This solution effectively controls machining errors and manufacturing costs by decomposing a single large-sized 2D dot array calibration board into two precisely machinable smaller ones. At the technical implementation level, it establishes spatial mapping relationships between the two calibration boards through optimized algorithms, ultimately completing angle calculations in a unified measurement reference board coordinate system.

The image pixel coordinate system $O_{1}\left(X^{\left(1\right)},Y^{\left(1\right)}\right)$ is defined with the top-left corner of the image as the origin $O_{1}$. The *X*-axis extends horizontally (along the image width), and the *Y*-axis extends vertically (along the image height), with both axes measured in pixels. The image physical coordinate system $O_{2}\left(X^{\left(2\right)},Y^{\left(2\right)}\right)$ uses the center of the image as the origin $O_{2}$. The *X*-axis and *Y*-axis align with the horizontal and vertical directions of the image, respectively, but their coordinates are measured in millimeters (mm). The 2D dot array calibration plate coordinate system $O_{3}(X^{\left(3\right)},Y^{\left(3\right)})$ takes the center of the top-left calibration circle on the calibration board as its origin $O_{3}$. The *X*-axis follows the long-edge direction of the board, while the *Y* axis aligns with its short-edge direction. The measurement reference plate coordinate system $O_{4}(X^{\left(4\right)},Y^{\left(4\right)})$ has its origin $O_{4}$ at point A, the fitted center derived from the four positioning holes $A_{11}$, $A_{12}$, $A_{21}$, $A_{22}$ at the plate’s top-left corner. The *X*-axis is defined by the line connecting point A to point B (the fitted center of the four positioning holes $B_{11}$, $B_{12}$, $B_{21}$, $B_{22}$ at the top-right corner). The *Y*-axis is defined by the line connecting point A to point C (the fitted center of the four positioning holes $C_{11}$, $C_{12}$, $C_{21}$, $C_{22}$ at the bottom-left corner).

#### 3.3.2. Calibration Method for Reference Plate

Let point A have coordinates $O_{i}(X^{\left(i\right)},Y^{\left(i\right)})$ in the pre-transformation coordinate system $A(X_{i},Y_{i})$ and coordinates $O_{i+1}(X^{\left(i+1\right)},Y^{\left(i+1\right)})$ in the post-transformation coordinate system $A^{\prime }(X_{i+1},Y_{i+1})$. Their relationship is governed by Equation (7) [40]:

(7) $$\left[\begin{array}{c} X_{i+1}\\ Y_{i+1} \end{array}\right]=\left[\begin{array}{c} \Delta X\\ \Delta Y \end{array}\right]+m\left[\begin{array}{cc} \cos\alpha & -\sin\alpha \\ \sin\alpha & \cos\alpha \end{array}\right]\left[\begin{array}{c} X_{i}\\ Y_{i} \end{array}\right]$$

In Equation (7), m is the scale factor, α is the rotation parameter of the coordinate system (i.e., the angle between the axis-$X_{i}$ and $Y_{i}$, with counterclockwise defined as positive), and the remaining are the translation parameters. To solve for the four parameters (ΔX,ΔY, m, α), the coordinates of two distinct feature points must be known in both the $O_{i}\left(X^{\left(i\right)},Y^{\left(i\right)}\right)\;$and $O_{i+1}(X^{\left(i+1\right)},Y^{\left(i+1\right)})$ coordinate systems.

Based on Equation (7), the conditional equations are formulated as follows:

(8) $$\left[\begin{array}{c} X_{i+1}\\ Y_{i+1} \end{array}\right]-\left[\begin{array}{c} X_{i}\\ Y_{i} \end{array}\right]=\left[\begin{array}{c} \Delta X\\ \Delta Y \end{array}\right]+\left[\begin{array}{cc} m\cos\alpha -1 & -m\sin\alpha \\ m\sin\alpha & m\cos\alpha -1 \end{array}\right]\left[\begin{array}{c} X_{i}\\ Y_{i} \end{array}\right]$$

Let a=mcos⁡α−1, and b=msin⁡α. Then,

(9) $$\left[\begin{array}{c} X_{i+1}\\ Y_{i+1} \end{array}\right]-\left[\begin{array}{c} X_{i}\\ Y_{i} \end{array}\right]=\left[\begin{array}{c} \Delta X\\ \Delta Y \end{array}\right]+\left[\begin{array}{cc} X_{i} & -Y_{i}\\ Y_{i} & X_{i} \end{array}\right]\left[\begin{array}{c} a\\ b \end{array}\right]$$

The equivalent expression is as follows:

(10) $$\left[\begin{array}{c} X_{i+1}\\ Y_{i+1} \end{array}\right]-\left[\begin{array}{c} X_{i}\\ Y_{i} \end{array}\right]=\left[\begin{array}{cccc} 1 & 0 & X_{i} & {-Y}_{i}\\ 0 & 1 & Y_{i} & X_{i} \end{array}\right]\left[\begin{array}{c} \Delta X\\ \Delta Y\\ a\\ b \end{array}\right]$$

where $L=\left[\begin{array}{c} X_{i+1}\\ Y_{i+1} \end{array}\right]-\left[\begin{array}{c} X_{i}\\ Y_{i} \end{array}\right]$, $B=\left[\begin{array}{cccc} 1 & 0 & X_{i} & -Y_{i}\\ 0 & 1 & Y_{i} & X_{i} \end{array}\right]$, $X=\left[\begin{array}{c} \Delta X\\ \Delta Y\\ a\\ b \end{array}\right]$, $P=\left[\begin{array}{cccc} 1 & 0 & 0 & 0\\ 0 & 1 & 0 & 0\\ 0 & 0 & 1 & 0\\ 0 & 0 & 0 & 1 \end{array}\right]$.

According to the least squares principle, the parameter vector X is calculated as follows:

(11) $$X=(B^{T}PB{)}^{-1}B^{T}PL$$

After obtaining the solution for X, the scale factor m and rotation angle α can be derived from parameters a and b using the following relationships:

(12) $$\alpha =\arctan\frac{b}{a+1}$$

(13) $$m=\sqrt{(a+1{)}^{2}+b^{2}}$$

Through the above calculation process, the calculations from the image pixel coordinate $O_{1}(X^{\left(1\right)},Y^{\left(1\right)})$ to the image physical coordinate system $O_{2}(X^{\left(2\right)},Y^{\left(2\right)})$, from the image physical coordinate system $O_{2}(X^{\left(2\right)},Y^{\left(2\right)})$ to the 2D dot array calibration plate coordinate system $O_{3}(X^{\left(3\right)},Y^{\left(3\right)})$, and from the 2D dot array calibration plate coordinate system $O_{3}(X^{\left(3\right)},Y^{\left(3\right)})$ to the measurement reference plate coordinate system $O_{4}(X^{\left(4\right)},Y^{\left(4\right)})$ are completed. Finally, we get the four parameter values from the coordinate system of the 2D lattice calibration plate to the coordinate system of the measurement reference plate so as to complete the calibration of the measurement reference plate.

## 4. Experiment and Verification

### 4.1. Experimental Setup

**Figure 5:**
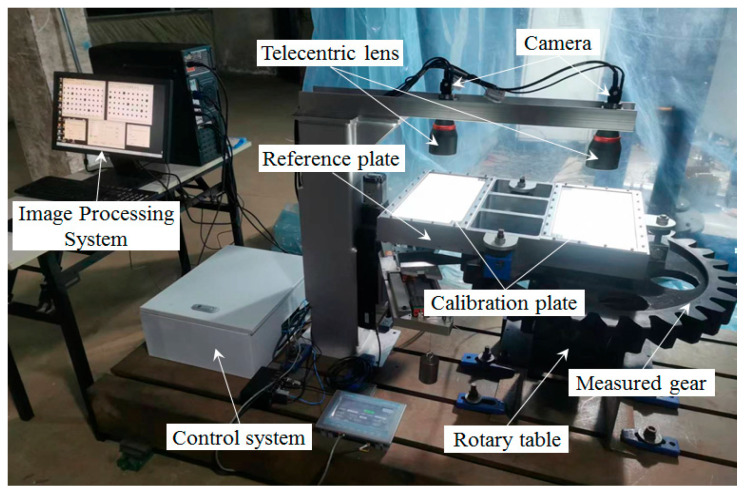
Experimental device.

The experiment utilized a large gear with a module of 20 and 30 teeth as the test object, applying the proposed method for angle rotation measurement. The vision measurement unit comprised: a CMOS monochrome camera (Photron, MV-E EM HS, Tokyo, Japan) with a resolution of 2248 × 2048 pixels; a bi-telecentric long-focus lens (Opto Engineering, TC12 48, Mantova, Italy) with a focal length of 131.4 mm; an LED collimated white light source (Jenoptik, JL-BRPX-380 × 180W-C, Jena, GermanyJ); and a digital light intensity controller (Jenoptik, JL-APS2-14424-2, Jena, Germany) to construct the angle measurement system. To validate the accuracy of the setup, the rotary stage (Newport, GTN-250R, Irvine, CA, USA) integrated with the gear was selected as the reference standard. This stage employs standard pulse signals for angle control and utilizes an internal rotary optical encoder grating as the traceable angular measurement standard, enabling real-time acquisition of the rotated angle values. The large gear was rigidly coupled with the high-precision rotary stage, and the experimental configuration is illustrated in Figure 5.

### 4.2. Experimental Environment Control

In terms of temperature control, considering the sensitivity of the CCD camera and the 2D dot-matrix calibration plate to temperature variations, fluctuations may lead to issues such as sensor thermal noise, lens and material thermal expansion, etc. The experimental environment temperature is strictly maintained within the range of 20 ± 2 °C. For vibration conditions, the setup strictly adheres to the ISO 20816-1 standard [41], ensuring that environmental vibration levels remain below 1.0 mm/s RMS. All data acquisition is triggered only when the vibration monitoring system confirms that the readings are within the safe threshold. Regarding lighting conditions, since the system is equipped with a digital light intensity controller, it can automatically adjust brightness based on the experimental site’s illumination, ensuring that the camera’s field of view remains clear at all times. Therefore, the actual lighting conditions impose minimal constraints. These environmental parameter control measures effectively ensure the reliability and repeatability of the experimental results.

### 4.3. Pixel Equivalent Calibration Experiment

**Figure 6:**
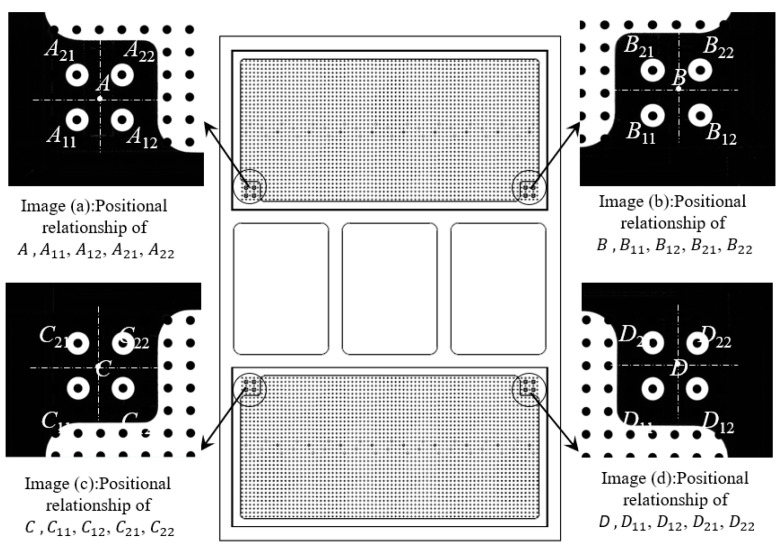
Calibration of reference plate.

In visual measurement, to obtain the dimensional parameters of an object, it is necessary to establish the correspondence between pixel dimensions and actual physical dimensions by calibrating the actual size represented by each pixel, which is referred to as the pixel equivalent [42,43]. The pixel equivalent serves as one of the most important parameters in visual measurement systems and has significant influence on measurement accuracy, thus requiring precise calibration [44]. To improve the measurement accuracy while considering the camera’s field of view and resolution, this study selected a 5 × 5 array of 25 calibration circles in the central region of a two-dimensional dot-pattern calibration board as the pixel equivalent calibration area, as illustrated in Figure 6. For the pixel equivalent calibration in visual measurement systems, this work adopted the method based on the center-to-center distance of calibration circles rather than the edge transition regions, mainly due to the following considerations. The edge transition regions of calibration circles exhibited obvious grayscale gradient variations, making them susceptible to image noise interference and highly sensitive to edge detection algorithms. In comparison, the center-to-center distance calibration method demonstrated significant advantages. The center coordinates were obtained through least-squares fitting of the entire circumference, which effectively suppressed the influence of local grayscale fluctuations at the edges. Experimental results showed that even when the edges of calibration circles exhibit blurring of up to ±5 pixels, the variation in center-to-center distance can be controlled within 0.1 μm, thereby ensuring the calibration precision.

The subpixel edges of calibration circles were acquired through edge extraction algorithms, followed by least-squares centroid fitting to determine the pixel coordinates of circle centers. The average pixel value of inter-centroid distances is derived by calculating the sum of pixel distances between the central centroid center and its 24 surrounding centroids. Given the known physical inter-centroid distance of 5 mm, the pixel equivalent of the measurement system was determined through the ratio of physical-to-pixel distances. The calibrated pixel equivalents of the dual-camera system are summarized in Table 1.

### 4.4. Calibration Experiment of Measurement Reference Plate

#### 4.4.1. Transformation from Image Pixel Coordinates $O_{1}(X^{\left(1\right)},Y^{\left(1\right)})$ to Image Physical Coordinates $O_{2}(X^{\left(2\right)},Y^{\left(2\right)})$

Processing image (a) in Figure 6 yielded the coordinates of the centers of four positioning holes ($A_{11}$, $A_{12}$, $A_{21}$, $A_{22}$) and the centers of calibration circles ($a_{11}$, $a_{22}$) approximately concentric with $A_{11}$ and $A_{22}$ within the image pixel coordinate system. Based on the pixel equivalent value derived from binocular camera calibration, the coordinates of $A_{11}$, $A_{12}$, $A_{21}$, $A_{22}$, and $a_{11}$, $a_{22}$ in the image physical coordinate system were calculated. According to the mean formula, the coordinate value of A in the physical coordinates of the image can be calculated. Similarly, processing images (b), (c), and (d) in Figure 6 yielded the results summarized in Table 2.

#### 4.4.2. Transformation from Image Physical Coordinates $O_{2}\left(X^{\left(2\right)},Y^{\left(2\right)}\right)$ to 2D Dot Array Calibration Plate Coordinates $O_{3}(X^{\left(3\right)},Y^{\left(3\right)})$

To determine the four transformation parameters ΔX, ΔY, m, and α for the coordinate system conversion between $O_{2}(X^{\left(2\right)},Y^{\left(2\right)})$ and $O_{3}(X^{\left(3\right)},Y^{\left(3\right)})$, two points were selected within the spatial regions containing points A, B, C, and D. These points’ coordinates in both the image physical coordinate system and the 2D dot array calibration plate coordinate system were computed. To minimize systematic errors during the transformation process and enhance calibration accuracy, diagonal points were selected for coordinate conversion, as illustrated in Table 3. The calculated values of the four transformation parameters (ΔX, ΔY, m, α) are summarized in Table 4.

**Figure 7:**
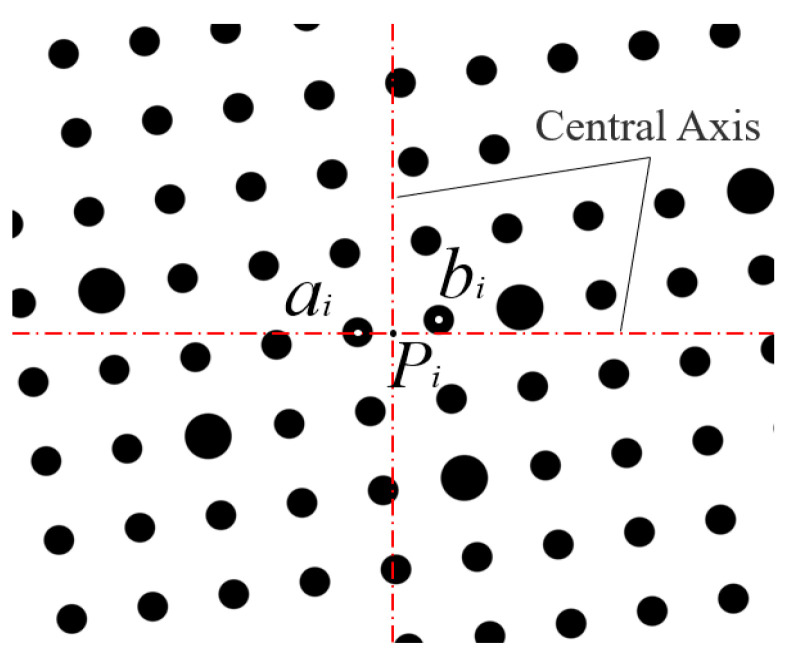
Coordinate position relationship of $a_{i}$, $b_{i}$, and $P_{i}$.

Using the coordinates of point A in the image physical coordinate system $A(X_{A}^{\left(2\right)},Y_{A}^{\left(2\right)})$ and the four transformation parameters (ΔX, ΔY, m, α), the coordinates of A in the 2D dot array calibration plate coordinate system $A(X_{A}^{\left(3\right)},Y_{A}^{\left(3\right)})$) can be derived. This iterative process was repeated for images (b), (c), and (d) in Figure 7 to calculate the respective four parameters and subsequently determine the coordinates of points B, C, and D in the 2D dot array calibration plate coordinate system. The parameters were specifically $B(X_{A}^{\left(3\right)},Y_{A}^{\left(3\right)})$, $C(X_{A}^{\left(3\right)},Y_{A}^{\left(3\right)})$, $D(X_{A}^{\left(3\right)},Y_{A}^{\left(3\right)})$. The calculated results are summarized in Table 5.

#### 4.4.3. Transformation from 2D Dot Array Calibration Plate Coordinates $O_{3}\left(X^{\left(3\right)},Y^{\left(3\right)}\right)$ to Measurement Reference Plate Coordinates $O_{4}(X^{\left(4\right)},Y^{\left(4\right)})$

The coordinates of the centers of 16 high-precision positioning holes ($A_{11}$–$A_{22}$, $B_{11}$–$B_{22}$, $C_{11}$–$C_{22}$, and $D_{11}$–$D_{22}$) on the measurement reference plate were measured using a Coordinate Measuring Machine (CMM) in the $O_{4}\left(X^{\left(4\right)},Y^{\left(4\right)}\right)$ coordinate system. The mean-value formula was applied to calculate the coordinates of points *A*, *B*, *C*, and *D* in the measurement reference plate coordinate system. The measurement results are listed in Table 6.

Using the coordinates of points *A*, *B* and *C*, *D* in both the 2D dot array calibration plate coordinate system and the measurement reference plate coordinate system, the four transformation parameters (ΔX, ΔY, m, α) for converting between the two coordinate systems were calculated. The results are summarized in Table 7.

### 4.5. Angle Rotation Measurement Experiment

#### 4.5.1. Angle Rotation Measurement Procedure

(1) The measurement reference plate was secured to the large gear using three magnetic mounts, and the plate was leveled via a three-point leveling device.
(2) Prior to initiating the measurement program, the initial coordinates of the calibration circle center closest to the field-of-view center and its right adjacent circle center were recorded in the 2D dot array calibration plate coordinate system: $a_{0}(X_{a_{0}}^{\left(3\right)},Y_{a_{0}}^{\left(3\right)})$, $b_{0}(X_{b_{0}}^{\left(3\right)},Y_{b_{0}}^{\left(3\right)})$.
(3) The rotary table was activated to rotate at a constant angular velocity via the measurement program while simultaneously triggering the binocular cameras for continuous image acquisition.
(4) The stereo camera system synchronously captures sequential images of the calibration target at fixed temporal intervals, generating dual image sequences $P_{i}(i=1,2,3...,n)$ and $Q_{i}(i=1,2,3...,n)$ from respective optical channels.

#### 4.5.2. Image Processing

The image processing methods for both cameras are identical. This section details the data processing procedure for Camera 1.

**Figure 8:**
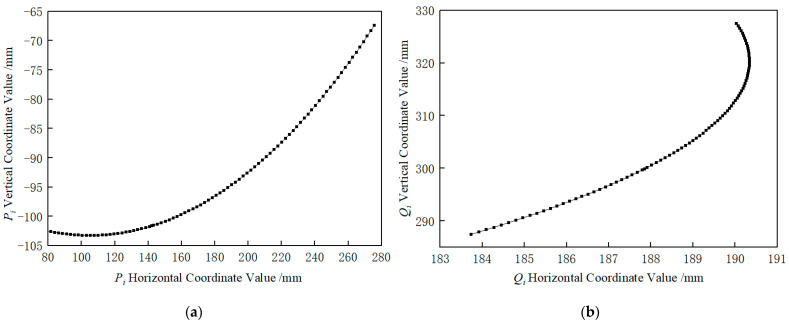
The coordinate trajectory of the camera center in the reference plate coordinate system. (**a**) The coordinate curve of Camera 1′s center point in the reference plate coordinate system. (**b**) The coordinate curve of Camera 2′s center point in the reference plate coordinate system.

(1) From image i, extract the coordinates of the calibration circle center closest to the field of view center and its right adjacent circle center in the image pixel coordinate system: $a_{i}\left(X_{a_{i}}^{\left(1\right)},Y_{a_{i}}^{\left(1\right)}\right)$ and $b_{i}(X_{b_{i}}^{\left(1\right)},Y_{b_{i}}^{\left(1\right)})$. Additionally, derive the camera center coordinate $P_{i}(X_{P_{i}}^{\left(1\right)},Y_{P_{i}}^{\left(1\right)})$ in the same coordinate system. Chose $a_{i}$ as the reference point because the area near the center of the field of view is less affected by camera distortion. Select $b_{i}$ primarily for programming convenience and ease of identification. The positional relationship of these three points is illustrated in Figure 7.
(2) Based on the pixel equivalent of Camera 1, compute the coordinates of the aforementioned three points in the image physical coordinate system: $a_{i}(X_{a_{i}}^{\left(2\right)},Y_{a_{i}}^{\left(2\right)})$, $b_{i}\left(X_{b_{i}}^{\left(2\right)},Y_{b_{i}}^{\left(2\right)}\right)$, and $P_{i}(X_{P_{i}}^{\left(2\right)},Y_{P_{i}}^{\left(2\right)})$.
(3) Given the initial coordinates $a_{0}(X_{a_{0}}^{\left(3\right)},Y_{a_{0}}^{\left(3\right)})$, $b_{0}(X_{b_{0}}^{\left(3\right)},Y_{b_{0}}^{\left(3\right)})$ of the calibration circle centers closest to the field-of-view center and its immediate right neighbor in the 2D dot array calibration plate coordinate system, iteratively compute the coordinates $a_{0}(X_{a_{0}}^{\left(3\right)},Y_{a_{0}}^{\left(3\right)})$, $b_{0}(X_{b_{0}}^{\left(3\right)},Y_{b_{0}}^{\left(3\right)})$ for each image frame using an 8-neighborhood coordinate extraction algorithm.
(4) Compute the four-parameter values $\Delta X_{i}^{\left(32\right)}$, $\Delta Y_{i}^{\left(32\right)}$, $\alpha_{i}^{\left(32\right)}$, and $m_{i}^{\left(32\right)}$ using the coordinates of points $a_{i}$ and $b_{i}$ derived from Steps (2) and (3) in both the image physical coordinate system and the 2D dot array calibration plate coordinate system. During rotational angle measurement, as the large gear undergoes rotary motion, the image physical coordinate system experiences relative rotation with respect to the 2D dot array calibration plate coordinate system. Consequently, the four parameters describing the coordinate transformation for each image i vary with each rotation angle.
(5) Using the four parameters ($\Delta X_{i}^{\left(32\right)}$, $\Delta Y_{i}^{\left(32\right)}$, $\alpha_{i}^{\left(32\right)}$, $m_{i}^{\left(32\right)}$) and $P_{i}(X_{P_{i}}^{\left(2\right)},Y_{P_{i}}^{\left(2\right)})$, compute the camera center coordinate $P_{i}(X_{P_{i}}^{\left(3\right)},Y_{P_{i}}^{\left(3\right)})$ in the 2D dot array calibration plate coordinate system.
(6) Transform $P_{i}(X_{P_{i}}^{\left(3\right)},Y_{P_{i}}^{\left(3\right)})$ to the measurement reference plate coordinate system via the pre-calibrated four-parameter set (ΔX, ΔY, m, α), resulting in $P_{i}(X_{P_{i}}^{\left(4\right)},Y_{P_{i}}^{\left(4\right)})$. The coordinate distribution of Camera 1 centered in this system is shown in Figure 8a.

Similarly, by repeating the above process for Camera 2 through continuous image acquisition, the coordinates $Q_{i}(X_{Q_{i}}^{\left(4\right)},Y_{Q_{i}}^{\left(4\right)})$ were derived and visualized in Figure 8b.

#### 4.5.3. Analysis of Rotation Angle Measurement Results

**Figure 9:**
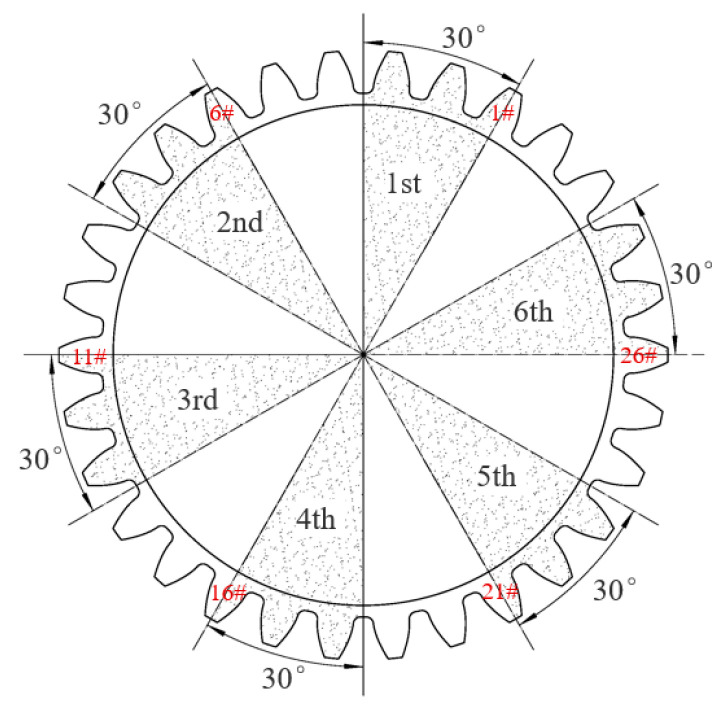
Rotation angle measurement positions (30° counterclockwise rotations starting from Gear 1, Gear 6, Gear 11, Gear 16, Gear 21, and Gear 26 respectively).

**Figure 10:**
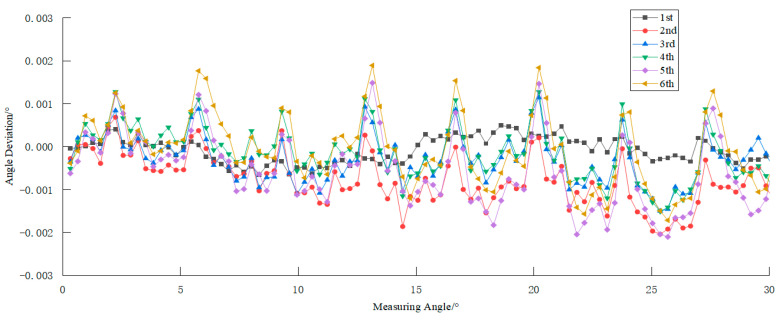
Verification results of measurement system accuracy.

Based on the coordinates of points $P_{i}\left(X_{P_{i}}^{\left(4\right)},Y_{P_{i}}^{\left(4\right)}\right)$ and $Q_{i}(X_{Q_{i}}^{\left(4\right)},Y_{Q_{i}}^{\left(4\right)})$ obtained through image processing and measurement procedures combined with the rotation angle measurement principle for large gears, the rotation angle can be calculated. Using the rotation angle values measured by the circular grating as the theoretical reference, the results obtained by this measurement method were compared and analyzed. The test object divided the measured large gear into six equal parts uniformly. Rotation angle measurements were conducted at each predetermined measurement position on the large gear, as shown in Figure 9. The system collected rotation angle data from each measurement point and plotted the rotation angle error distribution curve, as illustrated in Figure 10. The analysis of the experimental data revealed that the maximum error between the actual rotation angle and the theoretical rotation angle was 0.001895°, which was within 7 arcseconds.

**Figure 11:**
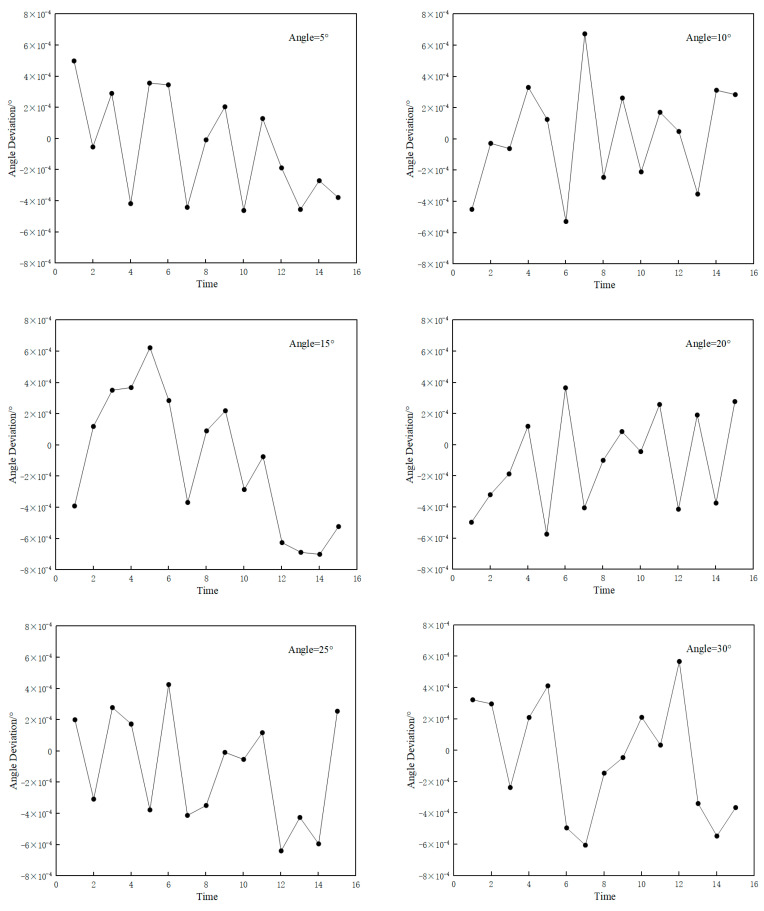
Verification results of repeatability measurement accuracy.

Under identical experimental conditions, the measurement system conducted 15 repeated measurements for six angular positions: 5°, 10°, 15°, 20°, 25°, and 30°. The repeatability deviation of any individual measurement was calculated as the difference between that specific measurement result and the arithmetic mean of the 15 measurement outcomes. As statistically demonstrated in Figure 11, the maximum observed repeatability deviation of the system reached 0.000699°, which corresponds to a repeatability precision within 3 arcseconds (3”).

#### 4.5.4. Angle Measurement Application

(1) Analysis of System Application Scenarios.

In the field of large gear manufacturing, high-precision measurement and online machining inspection are critical to ensuring gear performance. The dynamic angular measurement system for large gears, based on an extended visual field, achieves full coverage of gear end faces through dual-camera collaborative vision expansion technology. It can capture angular deviation of spur gears in real time, making it particularly suitable for low-speed, heavy-duty applications such as wind turbine gearboxes and marine propulsion systems. During online inspection, the system analyzes parameters like pitch error and tooth profile error using gear meshing measurement methods, providing direct feedback to machining tools for closed-loop compensation. This effectively addresses the latency issues associated with traditional offline inspection. The technology significantly improves consistency in batch gear production and enhances fault prediction capabilities, delivering core data support for intelligent manufacturing.

(2) Application Case of Rotation Angle Measurement.

**Figure 12:**
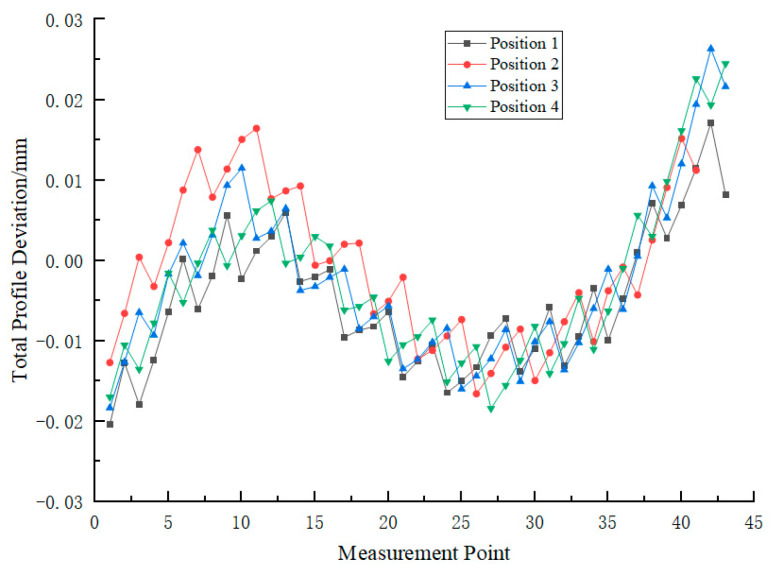
Measured value of total profile deviation for large gear.

In large gear profile measurement based on the meshing method, the measurement accuracy of the system is primarily determined by gear rotation angle error and meshing line error. Based on the experimental data from six angle measurements conducted within the 0° to 30° range and the displacement values measured by a laser rangefinder, the total profile deviation of the measured large gear was calculated to be 0.0401 mm, as shown in Figure 12.

According to GB/T 10095.1-2008 standard [45], for large gears with reference diameters ranging from 560 mm to 1000 mm and modules between 16 and 25, the allowable total profile deviation is 42 μm. Therefore, this system can meet the Grade 7 precision inspection requirements for large gears.

#### 4.5.5. System Optimization Prospects

The current measurement system employs a relatively bulky and fixed structural configuration. System optimization will be implemented through the following two aspects:

(1) Modular design concept.

Adopting a modular design philosophy to construct a reconfigurable vision measurement platform, the dual-camera setup and telecentric lenses are integrated onto an adjustable bracket. A composite bracket structure with multi-degree-of-freedom adjustment mechanisms ensures system stability while achieving spatial compression of the measurement system. Mechanical simulations are conducted to validate structural stability. Additionally, a retractable multi-functional reference plate is designed, with partitioned reuse of the reference plate to minimize space occupation.

(2) Intelligent adjustment.

For intelligent regulation, a machine learning-based adaptive zoom system is developed. Combined with closed-loop control algorithms, it enables a dynamic adjustment of working distance. An online calibration and compensation mechanism is established to maintain measurement accuracy. This optimization solution significantly enhances system compactness and environmental adaptability while preserving original measurement performance, providing technical support for industrial field applications.

## 5. Conclusions

To address the requirement for high-precision rotational angle measurement of a large gear, this study proposes a high-precision binocular vision-based measurement method. Sub-pixel edge extraction algorithms are employed to capture calibration circle contours, with least-squares fitting applied to determine circle center coordinates. The pixel equivalent ratio of the binocular camera is calibrated by comparing the physical and pixel dimensions of inter-circle distances. A measurement reference plate serves as the unified coordinate system benchmark, and its spatial calibration is achieved via a two-dimensional four-parameter transformation algorithm, ensuring multi-view data alignment in a global coordinate system.

The implemented binocular vision system dynamically captures calibration circle images of large gear to compute rotation angles in real time. The experimental results demonstrate that within a 0–30° rotation range, the system achieves a maximum absolute error of 0.001895° (equivalent to 7 arcseconds) and a repeatability deviation below 0.000699° (3 arcseconds). The system addresses the accuracy degradation in rotational angle measurement of large gear caused by mechanical wear or deformation in traditional contact-based sensors through non-contact measurement and is validated in demanding industrial scenarios requiring ultra-high precision, such as aerospace module assembly and wind turbine gearbox inspection.

## Figures and Tables

**Table 1:** Calibration results of pixel equivalent.

Camera	The Center Distance of Physical Dimensions/mm	The Center Distance of Pixel Dimensions/Pixel	Pixel Equivalent/μm
1	5	266.0175	18.7958
2	5	266.4734	18.7636

**Table 2:** Coordinate values of the selected points in the image physical coordinate system of the image.

Points	The Image Physical Coordinate Values	Points	The Image Physical Coordinate Values
$A_{11}$	(−7.7499, 4.5968)	$B_{11}$	(−7.7462, 3.5232)
$A_{12}$	(−7.7585, −5.3396)	$B_{12}$	(−7.7394, −6.4343)
$A_{21}$	(2.2464, −5.3572)	$B_{21}$	(2.1792, −6.4728)
$A_{22}$	(2.255, 4.6411)	$B_{22}$	(2.2253, 3.4502)
A	(−2.7518, −0.3647)	B	(−2.7703, −1.4834)
$C_{11}$	(−6.8633, 4.1014)	$D_{11}$	(−6.9554, 4.0799)
$C_{12}$	(−6.9426, −5.8532)	$D_{12}$	(−6.9522, −5.9734)
$C_{21}$	(3.1689, −5.8435)	$D_{21}$	(3.1409, −5.8716)
$C_{22}$	(3.0945, 4.1221)	$D_{22}$	(3.0282, 4.0708)
C	(−1.8856, −0.8683)	D	(−1.9346, −0.9236)

**Table 3:** The coordinates of the selected points in the image physical coordinate system and the calibration board coordinate system.

Points	The Image Physical Coordinate Values	The Calibration Board Coordinate Values
$a_{11}$	(−7.8233, 4.6096)	(−5, 170)
$a_{22}$	(2.1557, −5.4125)	(−15, 160)
$b_{11}$	(−7.7393, 3.6197)	(−360, 170)
$b_{22}$	(2.237, −6.4059)	(−370, 160)
$c_{11}$	(−6.9289, 4.1157)	(5, 15)
$c_{22}$	(3.0512, −5.9063)	(15, 5)
$d_{11}$	(−6.9941, 4.0914)	(360, 15)
$d_{22}$	(3.0367, −5.8802)	(370, 5)

**Table 4:** Four parameter values from the image physical coordinate system to the calibration board coordinate system.

Points	ΔX	ΔY	m	α
A	12.8328	165.4075	−0.9999	3.1394
B	367.7474	166.3997	−0.9999	3.1391
C	11.9367	10.8993	−0.9999	3.1395
D	366.9811	10.8884	−0.9999	3.1386

**Table 5:** The coordinates of A, B, C, and D in the calibration board coordinate system.

Points	The Calibration Board Coordinate Values
A	(10.08196, 165.0369)
B	(364.9811, 164.9096)
C	(10.05311, 10.02708)
D	(365.044, 9.970665)

**Table 6:** The coordinates of selected points in the reference plate coordinate system.

Points	The Reference Plate Coordinate Values	Points	The Reference Plate Coordinate Values
$A_{11}$	(−5.4162, 4.791)	$B_{11}$	(349.5118, 5.0201)
$A_{12}$	(−5.3551, −5.18)	$B_{12}$	(349.5381, −4.9659)
$A_{21}$	(4.5691, −5.1535)	$B_{21}$	(359.5017, −5.0561)
$A_{22}$	(4.5462, 4.7649)	$B_{22}$	(359.5675, 5.0564)
A	(−0.414, −0.1944)	B	(354.5298, 0.0136)
$C_{11}$	(−5.2281, 249.7988)	$D_{11}$	(349.7845, 249.9674)
$C_{12}$	(−5.2981, 239.7837)	$D_{12}$	(349.7289, 240.0091)
$C_{21}$	(4.6984, 239.7735)	$D_{21}$	(359.7011, 239.9157)
$C_{22}$	(4.7079, 249.7902)	$D_{22}$	(359.7338, 250.0244)
C	(−0.28, 244.7865)	D	(354.7371, 244.9792)

**Table 7:** Four parameter values from the calibration board coordinate system to the reference board coordinate system.

The Calibration Board	ΔX	ΔY	m	α
1#	−10.3268	−165.2615	−1.0003	3.1406
2#	−10.3247	234.7516	−1.0001	3.1409

## Data Availability

The raw data supporting the conclusions of this article will be made available by the authors upon request.
